# Clinical trial protocol to evaluate the efficacy of cefixime in the treatment of early syphilis

**DOI:** 10.1186/s13063-020-04885-z

**Published:** 2020-12-09

**Authors:** Shivani N. Mehta, Chrysovalantis Stafylis, David M. Tellalian, Pamela L. Burian, Cliff M. Okada, Carl E. Millner, Christopher M. Mejia, Jeffrey D. Klausner

**Affiliations:** 1 Department of Medicine, David Geffen School of Medicine at UCLA, Los Angeles, CA USA; 2 Department of Medicine, AIDS Healthcare Foundation, Los Angeles, CA USA; 3 Public Health Division, AIDS Healthcare Foundation, Los Angeles, CA USA

**Keywords:** Clinical trial, Cefixime, *Treponema pallidum*, Penicillin, Early syphilis, Syphilis

## Abstract

**Background:**

Syphilis rates have been increasing both in the USA and internationally with incidence higher among men-who-have-sex-with-men and people living with human immunodeficiency virus (HIV) infection. Currently, benzathine penicillin is the recommended treatment for syphilis in all patients. Global shortages and cost increases in benzathine penicillin call for alternative treatment options. This study evaluates the efficacy of oral cefixime for the treatment of early syphilis.

**Methods:**

We are conducting a randomized, multisite, open-label, non-comparative clinical trial in Los Angeles and Oakland, CA. Eligible participants are ≥ 18 years old, with primary, secondary, or early latent syphilis (rapid plasma reagin [RPR] titer ≥ 1:8). Patients with HIV infection must have a viral load *≤* 200 copies/mL and CD4+ T cell count ≥ 350 cells/μL during the past 6 months. Participants are randomized to receive either 2.4 M IU benzathine penicillin G intramuscularly once or cefixime 400 mg orally twice a day for 10 days. Participants return at 3, 6, and 12 months post-treatment for follow-up RPR serological testing. The primary outcome is the proportion of participants who achieve ≥ 4-fold RPR titer decrease at 3 or 6 months post-treatment.

**Discussion:**

Clinical trials evaluating the efficacy of alternative antibiotics to penicillin are urgently needed.

**Trial registration:**

Clinicaltrials.gov NCT03660488. Registered on 4 September 2018.

## Administrative information

The order of the items has been modified to group similar items (see http://www.equator-network.org/reporting-guidelines/spirit-2013-statement-defining-standard-protocol-items-for-clinical-trials/).

**Table:** 

Title {1}	Clinical Trial Evaluating the Clinical Efficacy of Cefixime in Treatment for Early Syphilis
Trial registration {2a and 2b}.	ClinicalTrials.gov Identifier: NCT03660488 [1] All items from ClinicalTrials.gov registry can be directly found within the protocol.
Protocol version {3}	Version 9; 11.20.2019
Funding {4}	AIDS Healthcare Foundation research grant (Grant #20181796)
Author details {5a}	1. Department of Medicine, David Geffen School of Medicine at UCLA, Los Angeles, CA/United States of America 2. Department of Medicine, AIDS Healthcare Foundation, Los Angeles, CA/United States of America 3. Public Health Division, AIDS Healthcare Foundation, Los Angeles, CA/ United States of America
Name and contact information for the trial sponsor {5b}	AIDS Healthcare Foundation
Role of sponsor {5c}	The sponsor played no part in study design; and will play no part in the collection, management, analysis, and interpretation of data; writing of the report; and the decision to submit the report for publication.

## Introduction

### Background and rationale {6a}

Syphilis rates have been increasing both in the USA and internationally. Between 2013 and 2017, the rate of syphilis cases in the USA increased from 17.9 cases per 100,000 (56,485 reported cases) to 31.2 cases per 100,000 (101,584 reported cases). Nearly half of the new early cases of syphilis occur among men-who-have-sex-with-men, while incidence is also high among and people living with human immunodeficiency virus (HIV) infection. Globally, there are 6 million new syphilis cases each year among persons aged 15–49 years globally, making syphilis an urgent global health threat [2–4]. Currently, benzathine penicillin is the recommended treatment for syphilis in all patients, including those living with HIV infection. Doxycycline and tetracycline are available alternative treatments for non-pregnant patients who are allergic to penicillin [5–8]. Injectable daily ceftriaxone is another alternative treatment that may be considered and is safe in pregnancy, as a recent review from our team showed [9].

Existing alternative treatment recommendations are based on clinical experience, a limited number of small clinical trials, and case series [5–7, 10]. However, each regimen poses clinical challenges. Doxycycline/tetracycline requires 14 days of treatment by mouth, with tetracycline requiring four daily doses. Ceftriaxone is administered intramuscularly, just like penicillin, but it requires daily injections for 10–14 days, making adherence potentially problematic. In pregnancy, only benzathine penicillin is recommended due to potential toxic effects of the alternatives or due to insufficient efficacy data (WHO). Shortages of benzathine penicillin worldwide have led to the use of unproven non-penicillin alternatives [10–12].

Considering the high cost and time required for developing and approving new antibiotics that can treat syphilis in patients with and without HIV infection, a new approach for identifying new, safe, and efficacious antibiotic treatments for syphilis is necessary. Previously Food and Drug Administration (FDA)-approved antibiotics that are safe and efficacious in other infections and have a favorable pharmacologic profile suggesting activity against *Treponema pallidum* may be effective alternatives for treating syphilis.

Cefixime is an FDA-approved orally administered third-generation cephalosporin with spectrum of activity and pharmacokinetic profile similar to that of ceftriaxone, a drug which has been used for the treatment for syphilis [13]. Cefixime is clinically used for uncomplicated urinary tract infections, upper respiratory tract bacterial infections, and in the treatment of uncomplicated *Neisseria gonorrhoeae* genital infection [14]. To our knowledge, it has never been studied as a treatment for early syphilis.

Cefixime has a well-studied pharmacokinetic profile [14–19]. Unlike other alternatives for syphilis, adverse event profiles are favorable with cefixime in non-pregnant as well as pregnant patients [20, 21]. Nearly 40–50% of the dose is absorbed when it is given orally, whether administered with or without food. Peak concentrations occur between 2 and 6 h following oral administration of a single 400 mg tablet. A single 400 mg tablet produces an average peak concentration of approximately 3.7 μg/mL (range 1.3–7.7 μg/mL). Typical blood levels of cefixime after a single dose of cefixime 400 mg by mouth are 4.84 μg/mL maximum at 4 h and above 1.0 μg/mL at 12 h. Serum protein binding is concentration independent with a bound fraction of approximately 65%. Cefixime is moderately distributed into extracellular water/tissue pools. Its half-life averages to 3–4 h but may range up to 9 h in healthy volunteers. Approximately 50% of the absorbed dose is excreted unmodified in the urine within 24 h and nearly 10% is excreted in bile [10].

We therefore believe that cefixime’s pharmacokinetic similarity to ceftriaxone and its safety in the treatment of pregnant women could potentially make it a viable option in the treatment of early syphilis.

### Objective {7}

The primary objective of our study is to determine the efficacy of cefixime 400 mg, taken orally two times a day (BID) for 10 consecutive days. Our hypothesis is that cefixime would be an efficacious treatment for early syphilis.

### Trial design {8}

This is a randomized, open-label, non-comparative pilot clinical trial. Participants will be randomly assigned (1:1 allocation) to receive either the standard of care benzathine penicillin injection or 10-days of oral cefixime. The study will require 2.5 years to be completed and each participant will be part of the study for 1 year. This pilot study could set the foundation for a larger randomized clinical trial evaluating the clinical efficacy of oral cefixime versus benzathine penicillin for the treatment of early syphilis.

## Methods: Participants, interventions, and outcomes

### Study setting {9}

The study will take place in 3 primary care HIV healthcare clinics and 1 wellness center of the AIDS Healthcare Foundation in Los Angeles and Oakland, CA. Healthcare clinics offer HIV and sexually transmitted infection (STI) primary care services while wellness centers are walk-in comprehensive sexual health clinics that offer HIV/STI screening services, STI treatment, and other prevention services.

### Eligibility criteria {10}

The inclusion criteria are:

1. Clinically or laboratory-confirmed new cases of early syphilis (primary, secondary, early latent syphilis) with a plasma rapid plasma reagin (RPR) ≥ 1:8
2. Eighteen years of age or older, capable of providing informed consent
3. HIV infected individuals must have CD4 T cell count ≥ 350 cells/mm^3^ and be virologically suppressed (viral load < 200 copies/mL) during the past 6 months
4. Able to travel to clinic once a day or be available for phone calls or receive a text message for at least 7–10 days

The exclusion criteria are:

1. Allergy to cefixime or penicillin
2. Pregnancy or a positive pregnancy test
3. Serofast RPR titer (prior titer ≥ 1:8 without a history of 4-fold titer decline)
4. Recent (within the past 7 days) or concomitant antimicrobial therapy with activity against syphilis, namely azithromycin, doxycycline, ceftriaxone, or other beta lactam antibiotics (e.g., amoxicillin)
5. A medical condition or other factors that might affect their ability to follow the protocol

### Who will take informed consent? {26a}

Patients must provide written, informed consent before any study procedures occur (randomization, blood sample collection, treatment). Consent will be obtained by a study clinician in a private examination room. The clinician will explain the study goal, research procedures, participant rights, and obligations following the informed consent sheet. The participant will be able to ask questions and will be given adequate time to review the consent and decide if they wish to participate. After reading through the document, the participant will provide their signature if they would like to take part in the study.

### Additional consent provisions for collection and use of participant data and biological specimens {26b}

Patients will also sign a Health Insurance Portability and Accountability Act (HIPAA) release form allowing access to clinical and laboratory data, including their HIV test results.

## Interventions

### Explanation for the choice of comparators {6b}

This is a pilot, non-comparative clinical trial designed to collect preliminary efficacy data. It includes an “experimental arm” of participants receiving cefixime and a contemporaneous “control arm” of participants receiving benzathine penicillin. The study was not designed to be adequately powered to show a statistically significant difference in the efficacy between the penicillin and cefixime arms.

### Intervention description {11a}

Eligible participants who provide their consent are randomized to the two arms of the study. Initially, the study team collects demographic (age, gender, race, ethnicity, contact, sexual orientation) and clinical information (most recent RPR titer, CD4 T cell count, HIV viral load). A venipuncture blood sample is collected by trained clinic staff and it is sent to the laboratory for testing. Testing is conducted on serum using the Arlington scientific RPR test kit (Arlington, VA) [22]. Participants are randomly assigned to the two treatment groups. Participants assigned to the penicillin arm will receive 1 dose of 2.4 M IU benzathine penicillin G on the day of enrolment. Participants who are assigned to the cefixime arm will be given 20 capsules of oral cefixime 400 mg on the day of enrolment to take for the following 10 days. Study staff observed receipt of the first dose. Subjects in the cefixime arm will then be asked to return for a clinical assessment or have a phone call assessment with the study team member 2 weeks following enrolment.

Study staff will follow up with all participants at 3, 6, and 12 months. In each follow-up, participants are asked questions regarding symptoms, antibiotic use in the past 3 months and the number of sex partners with whom they had condomless sex in the past 3 months. A new venipuncture blood sample is also collected for RPR testing.

### Criteria for discontinuing or modifying allocated interventions {11b}

Participants may request to leave the study or they may be withdrawn due to study-related adverse events. If a subject is discontinued from study participation due to an adverse event, they will be evaluated by the study clinicians for the need of additional treatment for syphilis. Safety data will be collected on any subject who is withdrawn from the study.

Participants in both study groups may receive additional treatment with penicillin, if they show no response to treatment (stable or absence of 4-fold decline at 6 months).

### Strategies to improve adherence to interventions {11c}

To ensure retention of participants, follow-up visits will be scheduled to coincide with routine clinic appointments for HIV care or preventive sexual health appointments, which occur every 3 months. In addition, study staff will contact participants, either over the phone or via text message, before their scheduled follow-up appointment. Finally, participants will receive reimbursement for their time and transportation in the form of a gift card.

### Relevant concomitant care permitted or prohibited during the trial {11d}

Usual HIV care and treatment for the participant will continue throughout the trial. Concomitant antibiotic use during the participation in the study duration of the trial will be recorded.

### Provisions for post-trial care {30}

Once participants complete the study, they will be able to continue receiving clinical care from the clinics. Participants study records will be reviewed and if necessary, additional treatment for syphilis will be administered according to the standard of care protocol.

### Outcomes {12}

The primary outcome is the successful treatment of early syphilis by the 3- or 6-month follow-up. The participants’ RPR titer will be used as the primary measure of outcome. Successful treatment is defined as an equal or greater than 4-fold RPR titer decrease, from baseline to 3 or 6 months after treatment.

### Participant timeline {13}

**Fig. 1:**
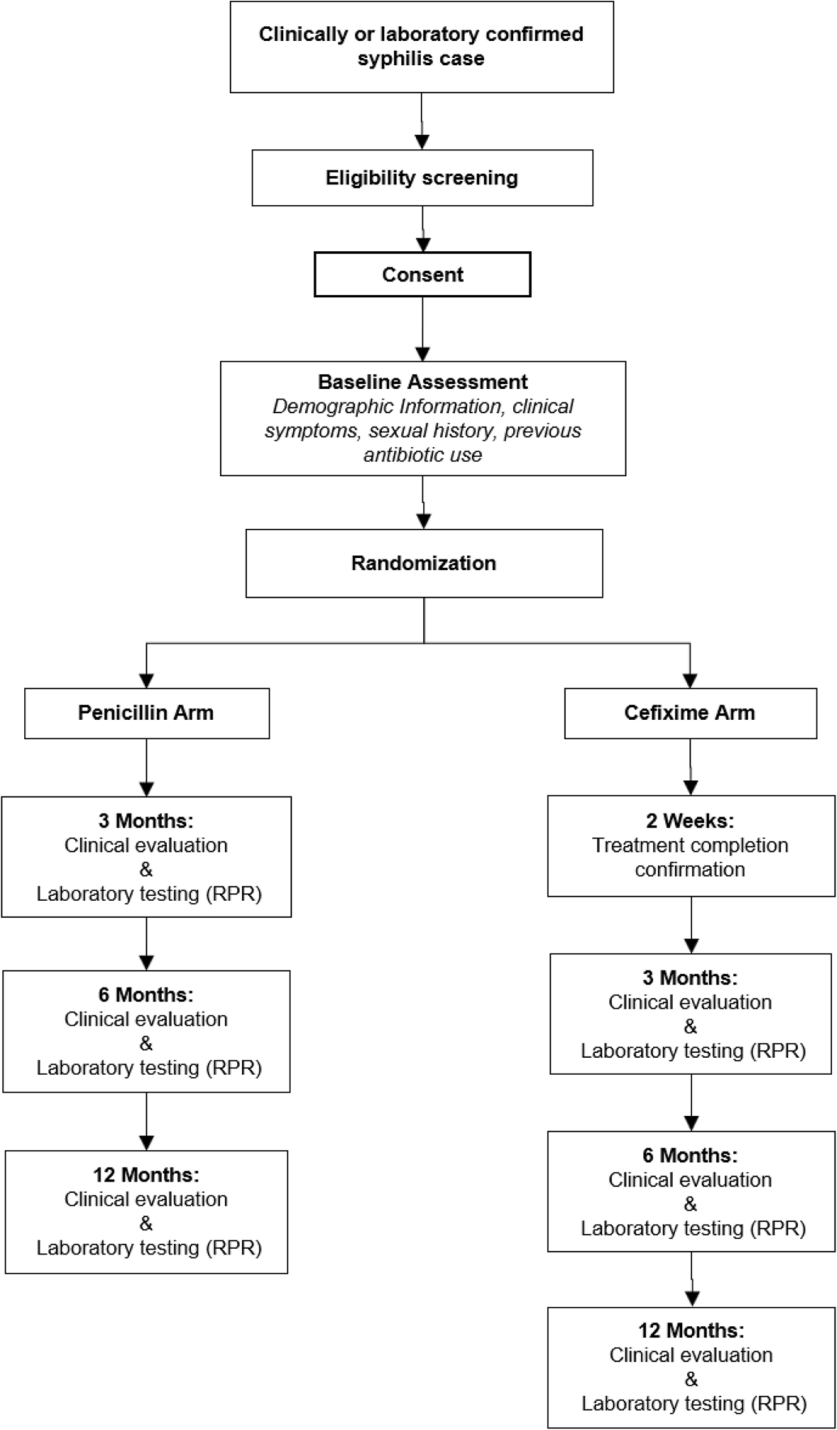
Participant enrolment and follow-up schedule

Participants will be part of the study for 12 months. Study evaluations will occur at 3, 6, and 12 months post-treatment. See Fig. 1 for the participant timeline for the trial and Table 1 for the study assessments.

**Table 1:** Study assessments by study time point

Assessment	Time point				
	Enrolment	10-day follow-up (cefixime arm only)	3-month visit	6-month visit	12-month visit
Eligibility screening	X				
Consent	X				
Randomization	X				
Demographic information	X				
Clinical & laboratory information	X				
Blood sample collection (RPR and RPR titer)	X		X	X	X
Treatment administration	X				
Evaluation of treatment completion		X			
Recent sexual history	X	X	X	X	X
Recent antibiotic use	X	X	X	X	X

### Sample size {14}

The primary outcome of the study will calculate the proportion of subjects with a 4-fold or greater decrease in RPR titer from baseline at 3 or 6 months in the per protocol analysis population.

Assuming a treatment success ratio of 90% among participants receiving cefixime, a sample size of 40 participants will yield a 95% confidence interval of (76–97%) (Table 2). Assuming 20% attrition due to loss to follow-up or non-compliance with the study medication schedule, enrolling 50 subjects will provide 40 evaluable subjects in the PP analysis population. Similarly, we will enroll 50 participants into the penicillin arm, as a contemporary cohort.

**Table 2:** Sample size and 95% confidence interval for successful treatment proportion of 90% and 95% among participants of the cefixime or penicillin arm

Sample size of evaluable subjects	Efficacy proportion of 90%	Efficacy proportion of 95%
20	68–99%	0.75, 1.0
40	76–97%	0.83, 0.99
60	79–96%	86–99%

95% confidence intervals calculated using a binomial exact test

### Recruitment {15}

Participant recruitment will occur in 4 AIDS Healthcare Foundation (AHF) Clinics based in Los Angeles, CA, and Oakland, CA. During the scheduled clinical visit, the study clinicians will approach cases of syphilis returning for treatment and they will provide a brief overview of the study. If the participant is interested, the clinician will determine patient eligibility. If the patient is eligible and agrees to participate, the clinician will conduct the informed consent and enrolment. Participants will be able to discuss study details with the clinicians and ask questions before signing the informed consent. Following enrolment, participants will be randomized, do their laboratory test, and receive the assigned treatment. Enrolment will be conducted by clinicians, who will be trained on the study procedures, and research assistants, who will assist in data collection, form preparation.

## Assignment of interventions: Randomization

### Sequence generation {16a}

After consent, participants are randomly assigned to the study arms with a 1:1 allocation. We will use a simple randomization method with a shuffled deck of sealed envelopes that contain a card with the assigned treatment. One hundred randomization cards, 50 for each study group, will be created before the initiation of enrolment, sealed in unmarked envelopes, and they will be distributed randomly to each of the study sites. No other factors will be taken into consideration for randomization.

### Concealment mechanism {16b}

The envelopes containing the randomization cards are sealed; thus, the team member conducting enrolment does not know the content of the envelope.

### Implementation {16c}

The study staff will ask the participant to select a sealed envelope from the shuffled deck. After selecting the envelope, the participant will reveal the treatment to themselves and the study staff.

## Assignment of interventions: Blinding

### Who will be blinded {17a}

This is an open-label clinical trial, and thus, neither the participants nor the study staff will be blinded to the assigned treatment. The data analyst performing interim and final statistical analyses will be masked to treatment assignment.

### Procedure for unblinding if needed {17b}

Not applicable.

## Data collection and management

### Plans for assessment and collection of outcomes {18a}

A venipuncture blood sample will be collected at 3-, 6-, and 12-month visits, and it will be tested for RPR titer. Study data collected on baseline include basic demographic information, sexual history, and laboratory tests (CD4 count, viral load, RPR titer). On each follow-up visit, sexual history, antibiotic use, symptoms, and the RPR titer will be collected. Data will be collected on paper data collection forms and will be entered into Research Electronic Data Capture (REDCap) [23, 24]. The paper data collection forms were specifically designed for our study by the study research team to capture data relevant to our objective. Data quality and completeness are ensured through a weekly internal review process in which the research assistants review all data collection forms for completeness and the database for missing information.

### Plans to promote participant retention and complete follow-up {18b}

Study follow-up visits are scheduled to coincide with routine clinic appointments within AHF. Study staff will send participants a 1-month, 2-week, and 1-day notification prior to their follow-up appointment.

### Data management {19}

Participant data will be collected on paper data collection forms and entered into REDCap. Data that will be entered into REDCap include participant information (name, date of birth, medical record number, contact information) and laboratory results.

### Confidentiality {27}

REDCap servers are encrypted, HIPAA-compliant, password-protected, and accessible only by designated study members. Hard copy data collection forms will be stored into a locked cabinet with limited access only to designated members.

### Plans for collection, laboratory evaluation, and storage of biological specimens for genetic or molecular analysis in this trial/future use {33}

Participants will provide a serum sample on baseline and at each follow-up visit. Samples will be collected by trained laboratory personnel inside the clinics. Samples will be sent to the laboratory within 24 h of collection. Sample processing and analysis will be conducted by trained laboratory staff following standard laboratory procedures. Samples will be destroyed after serological testing. The study team will not be involved in the sample collection, processing, analysis, and reporting.

There are no plans in this trial to evaluate or store biological specimens for genetic or molecular analysis for future use.

## Statistical methods

### Statistical methods for primary and secondary outcomes {20a}

The primary analysis for the main outcome will be conducted on the “per protocol” (PP) population. This will include participants who satisfy the inclusion and exclusion criteria, completed treatment (i.e., received the penicillin injection or received all of the cefixime pills), report no adverse events, returned for follow-up visits (3 and/or 6 months), and have an evaluable RPR result.

For each treatment group, we will calculate the proportion of PP participants who achieved a 4-fold RPR titer decrease at 3 or 6 months post-treatment (“treatment success”) and the exact binomial 95% confidence interval. Qualitative variables will be presented as frequencies with percentages and 95% confidence intervals (95% CIs) and quantitative variables as mean with standard deviation and range.

### Methods for additional analyses (e.g., subgroup analyses) {20b}

There will be no other additional analyses beyond the main analyses for the primary and secondary outcomes.

### Methods in analysis to handle protocol non-adherence and any statistical methods to handle missing data {20c}

Participants who do not adherence to the protocol or participants with missing data will not be included in the primary outcome analysis. We will be conducting an “intention to treat” (ITT) analysis that will include all individuals enrolled in the study, with evaluable RPR titter results and regardless of protocol non-compliance. For this analysis, missing data and non-adherent cases will also be considered as “treatment failure.” Similarly to the PP analysis, we will calculate the treatment success proportion for each treatment group among the ITT population at 3, or 6 months post-treatment and the exact binomial 95% confidence interval.

### Interim analyses {21b}

A summary of the enrolment progress, treatment success proportions, adverse events, and protocol deviations will be provided to the Data Safety Monitoring Board members.

### Plans to give access to the full protocol, participant level-data, and statistical code {31c}

The protocol of the study is publicly available on ClinicalTrials.gov (NCT03660488). Deidentified data will be available upon request to the study Primary Investigator.

## Oversight and monitoring

### Composition of the coordinating center and trial steering committee {5d}

The immediate study research team based in the University of California of Los Angeles meets on a weekly basis and oversees study recruitment, data quality, and study staff trainings. The immediate team is joined by a wider team of AHF study clinicians, based in study clinics, who also meet on a weekly basis.

### Composition of the data monitoring committee, its role, and reporting structure {21a}

The Data and Safety Monitoring Board (DSMB) will be composed of a physician, biostatistician, and regulatory affairs specialist/ethicist and will oversee the study throughout the 2-year study period. They will review study activities every 6 months. The committee will review safety data and clinical efficacy reports and determine whether it is clinically safe to continue the clinical trial. They will report their recommendation to the Primary investigator.

### Adverse event reporting and harms {22}

Cefixime and penicillin have a known safety and adverse event profile. Participants receiving penicillin may experience temporary mild pain on the site of injection. Cefixime can cause mild gastrointestinal reactions, such as diarrhea, loose stools, abdominal pain, and nausea [14]. Mild to severe allergic reactions are also expected in persons allergic to penicillin or cefixime.

Adverse events will be collected by spontaneous self-report. Study participants will be asked to report any problems throughout their participation to the study and specifically the duration of the treatment. We will use the Division of AIDS (DAIDS) Adverse Event grading system [https://rsc.niaid.nih.gov/clinical-research-sites/daids-adverse-event-grading-tables] that classifies adverse events by organ system and severity. Any AE/SAEs will be reported descriptively on the final project report and future publications.

The study site investigators will report serious adverse events and adverse events to the responsible IRB for that study site in accordance with respective IRB policies and procedures. Follow-up information to a reported adverse event will be submitted to the IRB as soon as the relevant information is available.

### Frequency and plans for auditing trial conduct {23}

The trial and individual clinic sites will be audited at least once during the duration of the study by the study sponsor (AIDS Healthcare Foundation).

### Plans for communicating important protocol amendments to relevant parties (e.g., trial participants, ethical committees) {25}

Amendments will be submitted to the IRB according to policies and guidelines. Any protocol changes will be promptly communicated to the IRB, the DSMB, and the study team.

### Dissemination plans {31a}

We plan to disseminate study results through peer-reviewed journal publications and conference presentations. Study findings will also be shared with relevant clinical and scientific groups.

## Discussion

This is a randomized, non-comparative, pilot study evaluating the efficacy of daily oral cefixime 400 mg for 10 days for the treatment of early syphilis. As syphilis rates increase and penicillin shortages continue to occur in the USA and worldwide, alternative treatments that are efficacious for both pregnant and non-pregnant populations, regardless of HIV status, are needed [6–8]. Already approved and antibiotics in clinical use with favorable pharmacokinetic profile, such as cefixime, should be clinically evaluated for alternative treatment options.

Data from this pilot study could be used as a foundation to assess the clinical effectiveness for cefixime in early syphilis treatment. Currently, our study is being conducted among non-pregnant individuals. However, subsequent clinical studies should also include women and pregnant women to address the gap in the treatment of maternal syphilis.

## Trial status

Recruitment was initiated on September 16, 2018, and will be complete on January 15, 2021. The current protocol version is version 9 (11/20/2020).
